# Access to health care perceived by parents caring for their child at home supported by eHealth—a directed approach introducing aperture

**DOI:** 10.1186/s12913-022-08398-0

**Published:** 2022-08-08

**Authors:** Mia Hylén, Stefan Nilsson, Inger Kristensson-Hallström, Gudrún Kristjánsdóttir, Pernilla Stenström, Rúnar Vilhjálmsson

**Affiliations:** 1 Department of Intensive and Perioperative Care, Skane University Hospital, Malmo and Lund, Sweden; 2 Department of Health Sciences, Faculty of Medicine, Lund University, Lund, Sweden; 3 Institute of Health and Care Sciences, Sahlgrenska Academy, University of Gothenburg, Gothenburg, Sweden; 4 University of Gothenburg Centre for Person-Centred Care (GPCC), Sahlgrenska Academy, University of Gothenburg, Gothenburg, Sweden; 5 Faculty of Nursing, School of Health Sciences, University of Iceland, Reykjavik, Iceland; 6 Department of Pediatric Surgery, Skåne University Hospital, Lund, Sweden; 7 Pediatrics, Department of Clinical Sciences Lund, Lund University, Lund, Sweden

**Keywords:** eHealth, Pediatric care, Access, Health care, Neonatal, Surgery

## Abstract

**Background:**

In recent years a variety of eHealth solutions has been introduced to enhance efficiency and to empower patients, leading to a more accessible and equitable health care system. Within pediatric care eHealth has been advocated to reduce emergency and hospital outpatient visits, with many parents preferring eHealth to physical visits following the transition from hospital to home. Still, not many studies have focused on access from the parental perspective. Therefore, the aim of the study was to analyze access to health care as perceived by parents when caring for their child at home, with conventional care supported by eHealth following pediatric surgery or preterm birth.

**Methods:**

Twenty-five parents who went home with their child following hospitalization and received conventional care supported by eHealth (a tablet) were interviewed in this qualitative study. Directed content analysis was used, guided by a framework for dimensions of access previously described as: approachability, acceptability, affordability, appropriateness, and availability.

**Results:**

All dimensions of access were present in the material with the dimensions of approachability, appropriateness and acceptability most frequently emphasized. The dimensions highlighted a strong acceptance of eHealth, which was perceived by the parents as beneficial, particularly access to communication with health care personnel familiar to them. The chat function of the tablet was often mentioned as positive. A new dimension was also identified: “aperture.” It is defined by the pathways by which communication is transmitted in cyberspace, and these pathways are not easily visualized for parents submitting information, therefore generating concerns.

**Conclusions:**

Parents generally experienced good access to the eHealth-supported health care. Describing access through its dimensions complemented previous descriptions of eHealth in pediatric care and gave new insights. As such, the new dimension of “aperture”, the indeterminate opening of pathways of communication reflecting the uncertainty of not comprehending cyberspace, could be further evaluated. The dimensional framework of access is recommended when evaluating eHealth in the future.

**Trial registration:**

ClinicalTrials.gov identifier: NCT04150120.

## Background

Access of individuals and groups to quality health care is a central principle in countries that have a socialized or a national health insurance system of care, such as the Nordic countries [1]. The provision of publicly accessible health care aims at promoting and protecting the health of the public and reducing inequalities in health [2].

In recent years, a variety of eHealth solutions has been advocated to improve access to professional health care, to enable patient self-management, and to deliver cost-effective care [3]. Pediatric care needs to be delivered in an accurate and timely manner [4]. eHealth is predicted to play a major role in pediatrics to help enable effective, accessible, and consumer-friendly care, not least during restrictions such as pandemics and other obstacles [5].

The use of eHealth to support a parent self-caring for their child at home has shown promising results [6], but research on eHealth’s impact on pediatric care and families’ experiences of access to health care has seldom been reported. Additionally, evaluation of eHealth from the perspective of parents [5] could improve our understanding of eHealth as a support for self-care within pediatric care.

eHealth is a broad concept that has been defined by WHO as “the transfer of health resources and healthcare by electronic means”, i.e. also including techniques such as telemedicine [6]. and is recognized as one of the fastest-growing tools used to improve access and quality of care [3, 6]. The area has been growing rapidly during the last decades and is by Eysenbach [7] broadly defined as not only a technical development in information delivered through the internet, but also an attitude to improve health care by using information and communication technology. Therefore, eHealth in this definition is more focused on the use of health and IT in health care, than the systems itself.

Since 2010, mobile applications have revolutionized communication both online and through computers, tablets, and phones. With a substantial increase in societal access to various eHealth applications, health care has become more convenient and less expensive [8, 9]. In a broader sense, eHealth can also be seen as a constructive framework to improve health care. Some of the beneficial impacts that are addressed in the discourse regarding eHealth are efficiency, patient empowerment, and encouragement for a new, more equal relationship between the health care and the patient [7].

Access to health care could be defined in different ways and from different vantage points. For example, it could be defined as a function of both supply and demand [10], or as the fit between the characteristics of the health care providers and characteristics and expectations of clients [11]. Access to care is said to be equitable when determined by need rather than other enabling or predisposing factors, such as education, income, insurance status, or geographic location [12]. Access concerns both the possibility of using services and the degree to which the care is used. In recent years, researchers have sought to disentangle and analyze more precisely the different dimensions of access to health care. An influential approach of this sort is the one presented by Levesque et al. [13]. The authors distinguish between five different dimensions of access: approachability, acceptability, affordability, availability, and appropriateness. Health care is approachable when individuals can identify services they need and know how to reach them. Health care is acceptable when provided in a manner consistent with individuals' values and norms, and it is affordable when individuals have time and resources to obtain the care. Health care is available when physically present and reachable in a timely manner, and it is appropriate when the services rendered fit and meet the needs of clients [13].

Systematic reviews still find few studies on the feasibility of eHealth in children’s health care and no studies especially address access to health care. The studies of parents that are available indicate an overall acceptance of eHealth within their child’s health care [14]. Supporting parents with tele homecare and videocalls (eHealth) has reduced emergency visits and hospital visits, and enhanced accessibility, with many parents in the experimental groups preferring eHealth to physical visits, not the least during the Covid-19 pandemic [14, 15].

Advanced pediatric surgery in Sweden has undergone a national centralization, leading to long geographic distances between the specialized pediatric surgery departments and patients’ homes. This could potentially present a risk to easy and immediate access to specialized care. This is especially the case in intensive neonatal care and advanced pediatric surgery that often involve close monitoring in the hospital. Less monitoring following the transition to home may increase risks to the children and decrease the sense of security for the families [16]. This raises the question of whether and how eHealth can strengthen the dimensions of access in the case of transitional pediatric care. This question has rarely been addressed, and never from the parents’ perspective.

## Aim

The aim of the study was to analyze dimensions of access to health care as perceived by parents when caring for their child at home, with conventional care supported by eHealth following pediatric surgery or preterm birth.

## Method

### Design

This qualitative study was based on parental interviews that were part of an intervention study (ClinicalTrials.gov identifier: NCT04150120). A specific mobile eHealth tablet was developed to allow digital communication between parents and health care providers during the family’s transition from hospital to home [15, 16]. The parents’ usage and experiences of eHealth for self-management, with a focus on an evaluation of the eHealth tablet, has previously been described by Lindqvist et al. [16]. In the present study, the previously described dimensional framework of access to health care [13] was used.

### Setting

This study was conducted between August 2019 and June 2020, thereby coinciding with the Covid-19 pandemic, with restrictions in place from March 2020 in Sweden. The children were cared for at the neonatal and pediatric surgery departments at a university hospital in the south of Sweden. The neonatal department has approximately 400 admissions each year and covers a catchment area of 500 000 residents who live within 80 km. The department of pediatric surgery is a national specialized center for specific malformations such as Hirschsprung’s disease, anorectal malformations, and esophageal atresia, and is a tertiary center for other specialized pediatric surgery. At the department 1200 operations are performed annually, including 150 advanced malformation procedures in neonates. It covers a catchment area of 5 million residents living within 600 km.

Families were included consecutively at each department after accepting an invitation to participate in the study. Inclusion criteria were parents or legal guardians who could read and write Swedish or English and had children under four years of age who were planned for discharge after advanced hospital treatment for prematurity or surgery for congenital colorectal malformations such as reconstruction of anorectal malformations and Hirschsprung’s disease. For families with a premature child, a home care team was automatically connected to the family at discharge, with routine visits planned according to gestational age and telephone counselling by the team. The families were also permitted to contact the neonatal department between 8 am and 4 pm. The conventional care following surgery included a one-week in-hospital stay postoperatively, with very early introduction of parent education in self-treatment of their child. After discharge the families were followed up by return visits and free telephone counselling with specialized pediatric contact nurses according to need.

In addition to conventional care, the parents were given an eHealth intervention delivered as an application on an Android-based tablet through which communication could be maintained with the professionals in the hospital [15]. The eHealth tablet operated by communicating wirelessly with a remote centralized server where all data were stored securely. Furthermore, the tablets could only be used for the purposes of this study and had no additional functions such as web browsers or games. Images sent were automatically deleted from the tablet and could therefore be seen retrospectively only by the health care professionals and not by the parents. The features of the tablet included a chat function (text messages) and video calls for follow-up meetings. The tablet also enabled photographs to be taken and transferred securely for review by the professionals. The parents made daily registrations of weight (prematurely born children), and reported the wellbeing and routine care of their child (reconstructive surgery) for the professionals at the hospital to see and follow up twice a day.

### Family characteristics

Background characteristics of the families is presented in Table 1. Twenty-five parents from 18 families were interviewed (both parents in seven families). Three of the families had pairs of twins, and so the total number of children was 21. The children from the neonatal department were preterm, born in week 33–35. Children from the pediatric surgery department were recovering after surgical procedures such as Transendorectal Pull-Through (TERPT) for Hirschsprung's Disease (HD), Posterior sagittal anorectoplasty (PSARP) for anorectal malformations, reconstructions for esophageal atresia through thoracotomies and laparotomy for appendicitis. The parents had the tablet at home for 2–5 weeks.

**Table 1:** Background characteristics of the 21 children and 25 parents from the 18 families included in the study

**Age (in years) and gender of interviewed parents**	
Female, n (%)	14 (56%)
Male, n (%)	11 (44%)
Age, median (range)	31 (28–42)
**Distance from home to hospital in kilometers, median (range)**	
All (18 families)	22.5 (8–365)
Neonatal department (9 families)	18.5 (8–60)
Pediatric Surgery department (9 families)	280.5 (20–365)
**Children´s age at hospital discharge**	
Neonatology (*n* = 12) Gestational age at discharge in weeks, median (range)	35 (34–36)
Pediatric Surgery (*n* = 9) Age at discharge in weeks, median (range)	5 (3–162)

### Data collection

Data were collected through semi-structured interviews. All interviews were conducted by the same researcher, external to the research project. The parents were contacted and asked for participation by e-mail, followed up by a telephone call, two to eight weeks after returning the tablet. Interviews were performed over the telephone and lasted 7–39 min (median 13 min). An interview-guide was used containing open questions regarding the parents´ experiences of going home with their child following discharge from the hospital, including their experiences of the eHealth tablet for communication with the health care providers. Both emotional and practical aspects of their experiences of caring for their child at home were brought up during the interview, and follow-up questions were used to gain depth and detail. The parents were also asked to describe the positive and negative aspects of using the eHealth tablet, along with thoughts on adaptation and improvements.

### Data analysis

Interviews with parents were analyzed qualitatively with directed content analysis as described by Hsieh & Shannon [17]. The dimensional framework of access to health care, adapted from Levesque et al. [13] was used to dissect accessibility to health care, as expressed by parents, following pediatric surgery or preterm birth.

Each dimension was described according to the dimensional framework. Two interviews were then analyzed independently by an interdisciplinary group consisting of three of the authors (the “analysis group”). Firstly, a discussion was held on how the dimensions should be interpreted until consensus was reached in the analysis group. Secondly, the results of the description were followed up by processing the domains, as well as coordination through discussion within the group of authors, until consensus was reached (Table 2).

**Table 2:** Dimensions of access to health care adapted from the framework by Levesque et al. [13]

Approachability	**Parents in need of health care can identify their need and are able to and do initiate contact with health services that could have an impact on their child´s health**
Acceptability	**Parents accept the care offered, including their role as recipients and providers of health care, including where, how, and what services are provided**
Affordability	**Parents have the time and resources to use service, and find the use of services worth the cost and effort**
Appropriateness	**The coordination and continuity of care initiated by the health services (through virtual and onsite contacts with known providers) generate a fit between services provided and the parent´s perceived needs**
Availability	**Health care is obtained and reached on site, either in the parent´s home or at the health care facility**

Further, the rest of the parental interviews were analyzed separately by the analysis group with frequent discussions during the process. If descriptions of access to health care were seen in the material that could not be classified using preexisting dimensions, they were saved for further analysis, either identified as a new dimension later or coded into the existing dimensions. The results of the independent individual classifications were compared and discussed within the analysis group. The principle decided beforehand was the majority rule, for example agreement between two out of three authors in the analysis group was needed for classification into a specific dimension.

Finally, the total coded material was divided into three parts and reviewed subsequently once more separately by members of the analysis group, before presentation and final confirmation by all authors. Both quantitative and qualitative analyses were used when reporting the result. Meaning units were first divided into predetermined categories and then calculated to map the representations of access for each dimension in 25 parent interviews.

### Ethical considerations

The study was approved by the Swedish Ethical Review Authority (no. 2019–0341). All participants received conventional care and were offered the eHealth tablet as a complementary component. Information regarding the study was given orally in a structured way followed by written information before written informed consent was obtained from the participants. Data were handled confidentially, and participating parents were able to quit without any explanation or effect on their care. Interviews and the data analysis were performed by researchers unknown to the parents and who had not been involved in the care of the children. One of the authors (PS), who was responsible for the surgical and medical care of the children undergoing pediatric surgery, was not involved in the data analysis, only in the writing stage.

## Results

All dimensions were represented in the analyzed material. As presented in Table 3, the dimensions of approachability, acceptability and appropriateness were more often present than affordability and availability. During the analysis a new dimension was also identified that could not be classified as any of the preexisting dimensions. This dimension was named aperture and identified as the indeterminate opening of pathways by which communication is transmitted in cyberspace, and these pathways are not easily visualized for parents submitting information. This created uncertainties regarding the possibility of trustworthy access to timely and effective care.

**Table 3:** Representations of access by each dimension in 25 parental interviews

*Dimension*	*No. of units*	*Units by access dimension*	*Interviews by access dimension*
**Approachability**	145	31%	100%
**Acceptability**	118	25%	92%
**Affordability**	39	8%	76%
**Appropriateness**	104	22%	92%
**Availability**	57	12%	84%
**Aperture**	12	2%	28%
Total	**475**	**100%**	

### Approachability

Approachability was referred to when the parents were able to identify their need for health care and initiate contact with health services. In other words, when the parents described seeking information during the care of their child at home and used different types of tools to achieve this support.

Compared with contacting care institutions unfamiliar with the child, parents experienced it to be more suitable to get in touch with health care professionals at the main treatment center who knew the child. Both the tablet and phone calls facilitated the ability to contact known health care professionals directly and reduced the need to go to hospital.

Although parents appreciated using the tablet, the technology did not always work perfectly. Sometimes they gave up and instead made a phone call or sent a text message. A phone call was sometimes perceived as a faster contact than using the tablet, especially if there was an urgent need. But phone calls were also perceived as imposing, as parents sensed that they were disturbing the health care professionals. The parents also experienced that it could be difficult to get in touch with the right person when they called. In these cases, the tablet was preferred because the health care professionals could respond when they had time.

The parents especially appreciated writing text messages to the health care professionals using the tablet when something had happened or when they wondered about something. The parents mostly thought that the health care professionals gave feedback quickly enough but the parents themselves did not always remember to read the answers on the tablet. The parents thought that a reminder function would have been helpful. The feedback from the health care professionals was experienced as instructive, and the possibility to ask and get answers over the tablet was reported to strengthen parents’ ability to self-care.

“So, I think I got answers, well I probably learned a lot through it (feed-back). Because as soon as I thought of something, I could ask. And that was really good.” (NI 24).

To get help and support, parents also sent pictures of their child’s operated areas. This facilitated their communication regarding their child’s skin problems connected to the operated area or healing processes. Using text messages and pictures was seen by parents as supportive in continuing their care at home. The parents appreciated being able to send pictures of eczema, for example, and being able to explain the color and structure of the baby's feces. When something looked strange and unknown, it was easier to send a picture showing what something looked like.

“Or there was a … a suture…that was…well…we thought it got a bit inflamed…eh a wound that was a bit, or a suture that we thought was a bit inflamed. And so we sent a picture. And then they wrote that we will talk to the physician and then they did talk to the physician.” (BKI 1).

The parents said that one of the tablet’s advantages was that it facilitated documentation and keeping track of their conversations with the health care professionals. The tablet was perceived as a valuable tool to be used both to inform health care professionals and to update the parents themselves. To be able to report easily to the health care professionals over the tablet empowered them to reflect on the child's health condition and progress in a secure way.

“And to just feel the security that they can also be there to observe and kind of… you could brainstorm with them. And you could, yourself, check how the statistics looked for weight gain and so forth… “(BKI 9).

### Acceptability

Acceptability refers to when parents were accepting of the care offered. It also included acceptance of their own involvement in the care of their child. Acceptability involved both the content of care as well as the form of care in question. In summary, parents welcomed the opportunity to stay at home with their fragile or ill child following hospitalization. The tablet provided them with continued communication with hospital personnel which provided security and helped them take care of their child's day-to-day needs.

Parents were generally favorably accepting of the services received when their child was in the hospital. The hospital was perceived as a secure environment with readily accessible professional services. Consequently, some parents had mixed feelings about leaving the hospital.

“Leaving the hospital ward is quite a change. Here you are constantly monitored. And then you go home. And then you are all alone with them [the child].” (NI 8).

However, the possibility of having regular contact with health professionals via the tablet, following the child's discharge from hospital, was seen as creating a sense of security and satisfaction with the available and provided care.

Getting a tablet from the hospital to take home for continued communication did not signify that parents could get immediate answers to all problems and concerns. Replies and responses from hospital personnel could sometimes take some time and it was not known in advance when replies would be forthcoming. This was generally accepted by the parents and recognized as reasonable, given other responsibilities held by the health care personnel.

“Of course, one would like them [the hospital personnel] to be in stand-by mode the whole time. And that they would answer immediately. But I understand that they … are also working on the hospital ward. “(BKI 9).

When explaining acceptability regarding the use of a tablet to communicate with health care professionals following the child's hospitalization, the participants pointed out that parents of young children are nowadays used to communicating electronically, meaning that in their generation this was easily accepted.

“I belong to the generation that is used to tablets, cellphones, and the like. So this [the tablet] was nothing new or strange. I just thought it was super.” (BKI 9).

The tablet was later returned by the parents, as they felt less reliant on it and had other ways of contacting health care personnel. The parents had become more confident in caring for their child at home and therefore more accepting of being without the tablet.

“…then we had come so far, and we didn't need it [the tablet] anymore. We kept it as long as we needed it. “(BKI 14).

Concerns involving acceptability were rare and related mostly to technical problems associated with using the tablet (such as battery life, sound, size of screen symbols, or picture quality) or concerns regarding security with sensitive information shared via the tablet.

### Affordability

Affordability refers to the willingness and capacity of individuals to spend resources and time using health services. It refers to the economic, social, and mental costs of obtaining care relative to the benefits. Responses relating to affordability in the parental interviews were invariably positive. Generally, the parents thought that compared with conventional health care, the tablet saved resources, effort and time when communicating and when receiving services on behalf of their child.

Using the tablet was also perceived as cutting down the number of trips to the hospital to access care. It was noted that the tablet could save on hospital visits which was good for everyone involved, meaning the child, the parents, and the hospital personnel.

“Instead of driving all the way if we don't need the physical contact, we could use the tablet or phone.” (BKI 24).

The ease of communication via the tablet was repeatedly mentioned as parents were able to write questions continuously as they came to mind and did not have to stay put to await answers. Several parents mentioned that the tablet was favored as a medium for interaction compared with telephone calls because the latter could be difficult and time-consuming. It was experienced that getting through and obtaining direct contact over the phone could be uncertain and therefore text messages on the tablet were favored.

“Calling them [the nurses] wasn’t very easy to call them … it wasn’t certain that they had time to answer immediately. Then it was easier to write a text message and then they would answer the same day. “(BKI 19).

However, not everyone thought that using the tablet was the most efficient communication medium. Some thought that it was sometimes easier to make phone calls, and some commented that an application on the mobile telephone would have been preferred instead of using the tablet.

### Appropriateness

Appropriateness refers to continued care that was initiated by the health services involving providers already known to the parents, meeting their perceived needs. Health services were perceived by the parents as appropriate when providing them with sufficient information as they cared for their child at home. Also, appropriateness was described as the structured information, requested through the tablet by the health care personnel, to be able to keep track of the child and support the parents at home with the intention of following up on the child's progress. This was supplemented with follow-up meetings, scheduled both at home through the tablet, and at the hospital. In general, parents perceived the health care providers to be welcoming regarding contacting them at any time, and open to their needs, and the tablets generated a pathway to direct contact that was appreciated.

In the neonatal eHealth care, routine information was asked for by the health services on a daily basis (weight, number of diaper changes, etc.). The purpose was perceived by the parents as supporting in caring for their child at home. The data (reported for follow-up) were entered into the tablet by the parents themselves. It was described in a positive way as a checklist or a regular report, visualizing the progress through measurements and graphs, thus helping them keep track.

A positive experience occurred when the entered information was noticed by health care professionals and generated attention and counter-questions. Entering these daily reports and getting responses to them in a formal way made the parents feel noticed, resulting in a feeling of safety and of being in control. However, it was also mentioned that the routine checkups could be experienced as impersonal if they only involved ticking boxes rather than using the free text or getting feedback.

“It became very illustrative really, to…to us. And surely for the healthcare personnel as well, that you could see, that the healthcare personnel could access graphs, weight curves, and those things.” (NI 6 + 8).

Furthermore, regular information gathered by health care personnel provided the parents with security and made them aware of the importance of a continued communication.

“Because it felt like they wanted to keep, how do you put it, the communication. So that you felt some form of… support and safety, I would say.” (BKI 24).

Appropriateness also referred to when the health services responded to parents´ questions in a timely and fast manner, taking time to meet their needs for information-giving, answers to medical questions, or support. However, times when the answer did not come as quickly as expected generated feelings of uncertainty and frustration, making the parents worried.

“And then, they answered fast and in a good way if there was anything the matter… and, also once we got to video chat with a physician.” (BKI 1).

Sometimes the communication was delayed due to technical issues such as the signal for new messages being low in volume or turned off, or the battery being low with messages being missed. In these cases, ability to communicate could vary daily – one day the video function was not working but the next day it was – making the parents feel uncertain of the technology.

### Availability

Availability refers to the extent to which health services are obtained and reached on site, either in the parent´s home or at the health care facility. To physically attain health care was described by the health care personnel paying the parents and children a visit in their own home. At times supplies needed refilling (syringes for example) in which case the parents needed to go to a pharmacy or to the hospital – but some even got the material sent to their house.

Sometimes the tablet did not generate pictures of sufficient quality for the health care providers to medically assess or resolve the child’s problem through the tablet. In these cases, the family needed to travel to the hospital. Still, the parents always felt welcomed by the health care professionals, and the child’s welfare was perceived as most important for both parties.

Having to transport the child for follow-up appointments at the hospital was described as challenging, especially when the travel took several hours. Travelling was described as burdensome and not beneficial to the child – contact over the tablet was then preferred. Also, during the pandemic, parents felt more secure if they could avoid places with other people because of possible infection.

“We had a newborn child and so it wasn´t ideal having to drive three times for four hours for a checkup. Especially in his condition. Since he needed a lot of stops to rest.” (BKI 6).

Home visits were more common within neonatal care and consisted mostly of outreach groups instigated by the hospital. Home visits were appreciated by the parents, and often concerned routine care such as height and weight measurements, but also practical issues such as replacing feeding tubes. Sometimes the visits were so frequent that it was perceived as being quicker to ask questions in person rather than through the tablet. On the other hand, home visits could be avoided when matters could be solved through the tablet instead; this was perceived as a positive outcome. Although it was positive to meet physically, the parents felt that some travel (for both parties) could be avoided especially during the pandemic.

### Aperture

Aperture refers to the opening of indeterminate pathways of communication that are transferred to cyberspace, where the roadmap is less defined. In contrast, the dimension of availability is a geographical road of transportation which is clear and visible to the parents.

This was reflected in uncertainty when a message or picture was sent digitally – the transmission, reception and recipient were not apparent and could not be easily visualized by the parents.

“I do not want for my child that there should be pictures of my child that are not suitable for anything other than a hospital.” (BKI 5).

Messages sent through electronic media were sometimes described as being sent into a vacuum. This led to uncertainties regarding the possibility of trustworthy access to care. The parents were concerned that sensitive photographs of the child´s body might have been left in an undefined place. Consequently, it was expressed that it is important to know what happens to the information that is transmitted in cyberspace.

“Taking photographs, of your own child, of such a delicate area and send to someone else. It doesn´t feel so good knowing that it could come into possession of anybody.” (BKI 13).

## Discussion

The study analyzed access to health care in terms of the proposed dimensions of access [13], and elucidated how eHealth solutions can affect parents' experiences of access to health care. However, these dimensions did not encompass all parental accounts of access to care. A new dimension appeared with content not fitting within other dimensions. We tentatively label this dimension aperture. It is defined by the pathways by which communication is transmitted in cyberspace, and these pathways are not easily visualized for parents submitting information. This contrasts with the availability dimension where access to health care is visualized through treatment sites, appointments, and transportation, and thereby tangible. The dimension availability is primarily interpreted in physical terms, either as care obtained by visits to hospital or in terms of home visits by health care professionals.

Aperture, as used here, refers to a space between realities, at the same time opening and constricting communication and the exchange of information in undetermined ways. Aperture includes the digital format, a rapidly emerging modality for access to health care. Sensitive information is always private in nature. Distributing such information via electronic media has been reported to potentially lead to serious threats to the individual [18]. Lack of security and privacy on the internet became apparent to many due to an incident where a UK consulting firm was able to collect data from 87 million Facebook users without their consent [19]. Although not directly comparable with health-related data, a vulnerability was revealed on the internet. The aperture dimension identified in our results is reflected by insecurities and threats when the parents reported not liking the idea of sending sensitive information about their child into the vacuum of cyberspace. For example, the parents felt insecure about who eventually got access to the pictures they took of their children and how they could be used. The dimension of aperture highlights the question of responsibility when it comes to eHealth. Health care services have an ethical responsibility to safely collect, use and share personal data [20]. Furthermore, the design and content of eHealth should be user-friendly and appropriate concerning support to the families so that the technology does not hinder the caring relationship [20, 21].

In this study, the dimension of acceptability was defined as parents accepting the care offered, and it included their role as recipients and providers of health care. Although, the parents felt safe at the hospital and had mixed feelings about leaving, they expressed that being at home was still their highest desire. Therefore, the possibility to go home and still have the support through the eHealth tablet was highly accepted and described as positive. The acceptance of being responsible as a provider of care with the support of eHealth is an issue that could be further explored among parents of children receiving health care.

With the adoption of the Convention on the Rights of the Child as law in Sweden, the best interests of the child should be a primary consideration in all aspects of health care. The Convention lays the foundation for a child-centred approach including children´s best interests in all decisions in health care involving them [22]. This study elucidated five original dimensions and a sixth new dimension of access to health care, and these six dimensions were able to encompass the scope of accessibility to health care relating to children's rights. To fulfil these rights, it is the health care professionals' responsibility to create care where parents can successfully handle a potentially unfamiliar situation in their home. The results indicated that the eHealth solution (tablet) in this study generally helped in this regard. The dimensions of approachability and appropriateness were more prominent in the analysis. These are the dimensions of access, along with acceptability, that are most strongly affected by the relationship and dialogue between health care professionals and parents about the care of the child and the safe and appropriate role of the parent. Approachability and appropriateness were more common than availability. Since the hospital-based health services in this study were physically present and obtainable, the fact that the parents often did not mention their availability was understandable.

People can be divided into “digital natives” and “digital immigrants” [23], with those born after 1980 defined as digital natives. This definition of those who have grown up with web-based solutions woven into everyday life refers to most of the participants in the present study. This is confirmed by some parents expressing that it felt obvious that they as young parents were offered the possibility of communication through digital solutions. Despite age differences, an Australian study showed that both digital natives and digital immigrants shared a skeptical attitude toward electronic exchange of health-related sensitive data between health care professionals and patients [24]. However, that study was conducted before the Covid-19 pandemic, which led to an increase in use of the digital format, dramatically changing the delivery of care [25]. Since then, eHealth has become further accepted as a format of standard care.

An important aspect of access in this study is that the parents were communicating through the tablet with professionals familiar to them, ever after their hospital care, and with whom they had a connection to prior to going home. In that sense, the eHealth solution became more personalized to the parents as they knew who was answering in the chat and on the video, and under which conditions. The need for a personal contact prior to an eHealth connection has previously been expressed by health care personnel. District nurses communicating through eHealth expressed that the physical encounter with patients prior to eHealth communication helped in building a trusting relationship which facilitated the use of eHealth [26]. The parents also got support and specialized preparation prior to going home with the tablet. This is an important aspect of eHealth for parents, as acknowledged in other studies [5]. It has also been highlighted that awareness and sensitivity to patients' needs and wishes is important when designing eHealth – for some there is still a need for physical follow-up meetings and other forms of contact such as phone calls [27]. Such awareness related to the target group is frequently expressed when customizing technology for eHealth [27, 28]. In view of the rapid growth of eHealth, the need for personalized access should be further explored to fulfil the families' rights to a suitable and effective care at home.

The children included in this study were young and needed their parents' support to further or secure their rights in decisions about care activities. Previously, it has been stressed that parents need support from health care professionals to be able to cope with the transition of care from hospital to home [29]. The dimensions approachability and appropriateness demonstrated that using an eHealth tool could facilitate this support, with the possibility of sending pictures to the healthcare professionals, for example. The possibility of health care professionals being able to see an image of a wound, for example, was appreciated by the parents, and it gave them confidence in taking the right decisions when caring for the child.

The dimension of affordability came up in positive terms in the parental interviews, indicating that the use of an eHealth tool reduced the parents' need to travel back and forth between home and hospital. Parents who used the tablet experienced savings in time and effort, and they said that care activities at home were affordable (i.e., worth the time, money, and energy that they spent). The use of home-based pediatric care has previously been presented as a safe and cost-effective, whether as a complement or an alternative to hospital care [30]. Cost-effectiveness in using eHealth has been identified in other age groups of patients, such as the elderly [31]. In addition, cost-effectiveness of eHealth has been demonstrated in other diagnoses, such as cardiovascular diseases [32] and diabetes [33]. However, it should be noted that some of the parents in this study thought that it was easier to make phone calls rather than use the tablet.

### Strengths and limitations

Five previously proposed access dimensions [13] generated both a structure and gave depth to the issue of access to care in interviews with parents who went home with their child following hospitalization. The dimensions of access described here, including the new dimension of aperture, reflected both positive and negative aspects of eHealth. The uncertainty towards electronic exchange of health-related sensitive data that was seen in the dimension of aperture is in line with previously described skepticism among digital natives [24].

As a possible limitation, the parents who accepted the invitation to participate in the study could already generally be more positive toward electronic media and therefore have a higher acceptance of eHealth solutions than those who chose not to participate. Also, only the parents’ perspective was considered in this study. Other perspectives left for further investigation are those of health care personnel and hospital management. At any rate, the implementation of an e-Health solution is not just a technical and managerial question. As Skär and Söderberg have noted [27], eHealth services should be developed and implemented based on the patient's and, in present study, child's specific needs to secure high-quality care.

Content analysis has emerged from a positivist research tradition with a quantitative description of the manifest content, which generates quantifiable units. Today, content analysis has also been inspired by a hermeneutic tradition [34]. A unique feature of content analysis is therefore the flexibility to be able to use both qualitative and quantitative methods, or a combination of these methods in data analysis. Consequently, the mixed method used is a strength in this article.

Further, to achieve rigor, an investigator triangulation was performed [35]. Independently reading and classifying the data before comparing and discussing within the analysis group aimed to achieve validity. Still, other method such as member checking could be used to assure that the result is in agreement with the informant´s experience [36].

## Conclusion

The findings of the present study indicate that parents in the transfer from hospital to home following preterm birth or pediatric surgery care generally experienced good access to the eHealth-supported health care that they were offered. The frequent mentioned dimensions approachability and appropriateness revealed that the parents reported high access to health care because they could easily initiate contact through the tablet. eHealth was perceived to generate a fit between a parent's needs and health care, exemplified by the fact that parents appreciated being able to stay in contact with familiar professionals at the hospital. The possibility of writing chat messages whenever a question arose, without having to make a phone call, was a very positive outcome. Health care professionals generally responded in a timely manner and the generated access supported parents’ confidence and control. However, if an urgent need arose which could not be met by the eHealth tablet, or if technical issues delayed communication, the tablet was immediately perceived as negative since other communications sources were then needed. At any rate, the pediatric care and the eHealth tablet were generally highly accepted by the parents, and eHealth communication was regarded by the current generation of parents interviewed here as a normal way to communicate.

The analysis of the study material supported by previously developed dimensions gave a structure and new insights into how access is perceived by parents. A new dimension was identified, aperture, which is an opening, or pathway, where communication is transmitted in cyberspace which is less defined and therefore generated concerns. This is a new aspect of access that has a close connection to the growing area of eHealth and should be explored further. When developing eHealth interventions, researchers should be well advised to include a qualitative participatory design, attentive to the needs and wishes of the target group.

## Data Availability

The data that support the findings of this study are available from Lund University, but restrictions apply to the availability of these data, which were used under license for the current study, and so are not publicly available. Data are however available from the "corresponding author" upon reasonable request and with permission of Lund University.

## Abbreviations

eHealth: Electronic Health
ICT: Information and communication technologies
